# Supplementary material for: Predation and fragmentation portrayed in the statistical structure of prey time series
Source: BMC Ecol. 2009 May 6;9:10. doi: 10.1186/1472-6785-9-10 (PMC2689204; doi:10.1186/1472-6785-9-10)
Supplement: Additional file 2 — Voles and related classes ODDox Documentation. ODDox documentation of the agent-based model (ALMaSS) applied by Hendrichsen et al. The documentation is started by activating main.html. [file 1472-6785-9-10-S2.zip › Vole_ODDox/pages.html]

ALMaSS ODDox: Page Index

- Main Page
- Related Pages
- Classes
- Files

# Related Pages

Here is a list of all related documentation pages:

- Crop Classes- Farm Management- References

---

Generated on Thu Jan 22 14:13:44 2009 for ALMaSS ODDox by 
 1.5.6
